# The net cost of incorporating resistance testing into HIV/AIDS treatment in South Africa: a Markov model with primary data

**DOI:** 10.1186/1758-2652-14-24

**Published:** 2011-05-15

**Authors:** Sydney Rosen, Lawrence Long, Ian Sanne, Wendy S Stevens, Matthew P Fox

**Affiliations:** 1 Center for Global Health & Development, Boston University, Boston, MA, USA; 2 Health Economics and Epidemiology Research Office, Wits Health Consortium, Johannesburg, South Africa; 3 Faculty of Health Sciences, University of the Witwatersrand, Johannesburg, South Africa; 4 Department of Molecular Medicine and Haematology, University of the Witwatersrand, Johannesburg, South Africa; 5 National Health Laboratory Services, Johannesburg, South Africa; 6 Department of Epidemiology, Boston University School of Public Health, Boston, MA, USA

## Abstract

**Background:**

Current guidelines for providing antiretroviral therapy (ART) in South Africa's public sector programme call for switching patients from first-line to second-line treatment upon virologic failure as indicated by two consecutive viral loads above 5000 copies/ml, but without laboratory evidence of viral resistance. We modelled the net cost of adding resistance testing for patients with virological failure and retaining patients without resistance on first-line therapy, rather than switching all failures to second-line therapy.

**Methods:**

Costs were estimated for three scenarios: routine maintenance (standard care without resistance testing, switch all failures to second line); resistance testing (resistance test for patients with failure, switch those with resistance); and limited testing (resistance test for patients with failure in the first three years, switch those with resistance). A Markov model was used to estimate the cost of each arm over five years after first line initiation. Rates of treatment failure, viral resistance and treatment costs were estimated with primary data from a large HIV treatment cohort at a public facility in Johannesburg. Future costs were discounted at 3%.

**Results:**

Virological failure rates over five years were 19.8% in routine maintenance and 20.2% in resistance testing and limited testing; 16.8% and 11.4% of failures in routine and limited testing, respectively, did not have any resistance mutations, resulting in 3.1% and 2.0% fewer patients switching to second-line ART by the end of five years. Treatment costs were estimated at US$526 and $1268 per patient per year on first-line and second-line therapy, respectively; a resistance test cost $242. The total average cost per patient over five years was $2780 in routine maintenance; $2775 in resistance testing; and $2763 in limited testing.

**Conclusions:**

Incorporating resistance testing into treatment guidelines in South Africa is potentially cost-neutral and can identify other reasons for failure, conserve treatment options, and generate information about emerging resistance patterns.

## Background

Research in South Africa suggests that a sizable minority of HIV/AIDS patients on antiretroviral therapy (ART) with detectable viral loads remain susceptible to first-line antiretroviral drugs, and are thus apparently failing treatment without evidence of drug resistance. In three cohort studies of adult patients receiving standard ART in KwaZulu-Natal and Gauteng provinces [1-3], 16.5%, 16.8% and 21.7% of patients with virological failure had no major resistance mutations, respectively.

South Africa's guidelines for adult ART call for patients to be switched from first-line to second-line therapy following virological failure [4]. Resistance testing is not mentioned in the guidelines and is not done in routine public sector care. Switching patients to second-line therapy when they are failing virologically but are not resistant to first-line drugs, however, is not likely to improve these patients' outcomes. It also prematurely restricts their future treatment options, while incurring the unnecessary cost of expensive second-line drugs. A recent analysis of the cost of second-line therapy in South Africa estimated that it is 2.4 times more expensive than first-line therapy per year in care [5].

We hypothesized that despite the relatively high cost of performing resistance assays, resistance testing for patients with detectable viral loads could prove to be cost-neutral or even cost-saving for the South African treatment programme if patients who do not have resistance are maintained on first-line drugs, rather than being switched to the more expensive second-line regimen. The objective of this analysis was to model the net cost of such a strategy, using data from a large public sector treatment facility in Johannesburg.

## Methods

### Model

**Figure 1:**
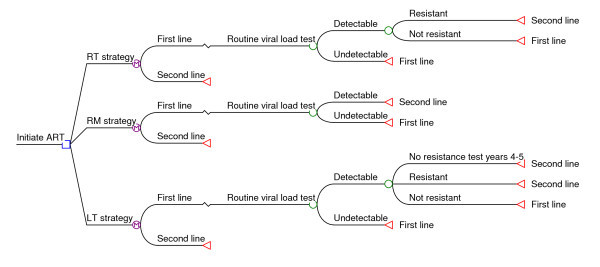
**Structure of decision model used in analysis**.

We developed a state-transition decision (Markov) model to estimate the costs of three strategies for switching patients from first- to second-line ART over the first five years after ART initiation under the national treatment guidelines in effect from 2004 to 2010 [4]. These guidelines were used because all model parameters are based on patient data accrued prior to 2010. The decision model, which was programmed in TreeAge Pro 2009 (TreeAge Software Inc., Williamstown, MA), is illustrated in Figure 1.

A hypothetical cohort of patients move through the model in six-month cycles, beginning with first-line ART initiation and continuing for a total of five years of follow up. A cycle length of six months was selected because routine viral load testing was performed every six months under the prevailing treatment guidelines. At the end of each cycle, a viral load test is done for all patients and a second confirmatory test for those whose first result is detectable (>1000 copies/ml). In the routine monitoring (RM) strategy, which reflects standard practice at the study site, all confirmed virologic failures are automatically switched to second-line therapy. In the resistance testing (RT) strategy, resistance testing is conducted at the end of the cycle for virological failures, and only patients with resistance to first-line drugs are switched to second-line therapy.

Finally, we hypothesized that the probability of failing virologically but not having resistance is greater during a patient's earlier years on ART and declines in later years. We therefore estimated the costs of a limited testing (LT) strategy, in which those with confirmed virological failure any time in their first three years on ART have a resistance test at the end of the cycle in which their second consecutive detectable viral load occurs and are managed as in the RT strategy, while those on ART longer than three years at the time of confirmed failure are managed as in the RM strategy.

In all scenarios, patients switched to second line remain on second line for the rest of the study period; patients still on first line at the end of each cycle continue to face cycle-specific probabilities of virologic failure in each remaining cycle. All patients remain alive and on either first- or second-line treatment at the end of the five-year modelling period.

### Study site

Modelling parameters for the study were estimated from the Themba Lethu Clinical Cohort, a population of approximately 10,000 adult ART patients at a previously described [6] public sector clinic in Johannesburg. Standard first-line therapy under the national treatment guidelines in effect until 2010 included stavudine or zidovudine (AZT), lamivudine, and efavirenz or nevirapine. Standard second-line therapy was AZT, didanosine, and lopinavir/ritonavir (LPV/r) [4]. Unlike in many other resource-constrained countries, monitoring of viral load is routine in South Africa and was called for every six months. Patients were switched to second-line therapy if viral load remained detectable in a consecutive, confirmatory test.

### Data

Rates of virological failure and non-resistance were estimated from the study site. Using the site's electronic medical record database (Therapy Edge™), the probability of virological failure per six-month interval on treatment was calculated for the first six intervals (three years) after first-line ART initiation, conditional on remaining alive and on ART at the site. The rate of virological failure in the last interval (months 25-36 on ART) was also applied to each six-month interval in years four and five of the model, as there were too few patients with this duration of follow up in the dataset to allow these values to be estimated from the treatment cohort. At this site, a threshold of 1000 copies/ml was used to define failure, and confirmatory viral load tests were done at intervals ranging from two weeks to several months after the first detectable result.

In a previously published study presenting pooled data from our study site and another large public sector treatment facility in Johannesburg, 16.8% of patients with two consecutive detectable viral loads were found to have no known mutations [2]. Time on ART prior to virological failure was not reported. We obtained most of the original data from our study site and re-estimated the rate of non-resistance over the first five years on ART. Our re-estimation generated a cumulative non-resistance proportion for the patients in our sample of 17.4%, close to the 16.8% value published for the two Johannesburg sites together. Because of very small sample sizes within each six-month interval on ART, we applied the cumulative rate to each interval, rather than varying it by interval. For the LT scenario, we used the same method to estimate the proportion non-resistant over the first three years on ART only.

Estimates of the annual unit costs of first- and second-line therapy were drawn from a published study conducted at the same site [5]. These estimates include ARVs, other drugs, laboratory tests, outpatient visits, and outpatient clinic fixed costs and infrastructure. Inpatient care is excluded. The cost of a resistance assay and viral load test was provided by the National Health Laboratory Service (authors' data). All costs are shown in US dollars after applying the 2008 average exchange rate of 8.28 South African rands to one US dollar [5]. An annual discount rate of 3.0% was applied to future costs.

### Sensitivity analysis

To examine the sensitivity of our results to uncertainty in the model transition probabilities (rates of virological failure and non-resistance among failures), we conducted a probabilistic sensitivity analysis (Monte Carlo simulation) using beta distributions for the cycle-specific virological failure rates and the cumulative non-resistance rate. Beta distributions were created with the alpha parameter equal to the total sample size minus the number of events observed and the beta parameter equal to the number of events. We then used the 2.5^th ^and 97.5^th ^percentile of the simulated distributions to create 95% simulation (uncertainty) intervals (SIs) for our point estimates.

In addition, we conducted a series of one-way sensitivity analyses for the three main cost inputs to the model: the cost of a year of first-line treatment; the cost of a year of second-line treatment; and the cost of a resistance assay. For the treatment costs, we considered the impact of increasing or decreasing the cost per year by 20%. For the cost of a resistance test, which may vary more widely from setting to setting, we considered a cost increase or decrease of 50%.

Access to anonymized patient-level data was approved by the Institutional Review Boards of Boston University and the University of the Witwatersrand.

## Results

### Parameter values

Data for 8500 patients from the Themba Lethu Clinical Cohort were included in the analysis of virological failure rates. Overall, 8.4% of patients in the sample had two consecutive detectable viral loads. The proportions of first-line patients with virological failure per six-month interval on ART are shown in Table 1. After the first six months on treatment, failure rates averaged 2% to 3% per cycle.

**Table 1:** Virological failure and non-resistance rates used in the analysis

Parameter	n	Value (95% CI)
Virological failure rates (2 consecutive tests >1000 copies/ml)		
6 months	8500	0.92% (0.71%-1.12%)
12 months	6979	2.99% (2.59%-3.39%)
18 months	5782	2.77% (2.34%-3.19%)
24 months	4672	2.35% (1.92%-2.79%)
30 months	5094	2.00% (1.62%-2.39%)
36 months and remaining six-month intervals	3569	2.16% (1.68%-2.63%)
Non-resistance rate among virological failures	109	17.4% (10.31-24.55%)

Resistance test results, as defined by Wallis *et al *[2], and time on ART were available for 109 patients with virological failure. Most (n = 98, 90%) had been on ART for six to 36 months at the time of testing, but a few (n = 2, 2%) were in their first six months of therapy and the rest (n = 9, 8%) had initiated treatment 36 to 60 months before. Of the 109 with virological failure, a total of 19 (17.4%) did not have any major resistance mutations. Most of these (74%) experienced virological failure between 18 and 36 months on ART. No instances of non-resistance were found among patients on ART for more than 42 months, though the sample size for this duration on treatment was very small.

Table 2 indicates the cost and other input parameters used in the analysis. Second-line therapy costs nearly two-and-a-half times that of first-line therapy, as mentioned, while a resistance test costs the equivalent of nearly half a year of first-line therapy.

**Table 2:** Cost parameters used in the analysis

Parameter	Value	Lower value for sensitivity analysis	Upper value for sensitivity analysis
Unit costs (2008) USD			
First-line ART per patient per year	$526*	$421	$631
Second-line ART per patient per year	$1268*	$1014	$1522
Resistance test	$241.55^†^	$120.78	$362.33
Viral load test	$36.23^†^	n.a.	n.a.
Discount rate applied to future costs (annual)	3.0%		
Exchange rate (Rand/$)	8.28^‡^		

*Long *et al*, 2010 [5]

^† ^Authors' data from National Health Laboratory Service, South Africa

^‡^Exchange rate taken from source [5]; average daily exchange rate in 2008

### Model output

Table 3 reports the cumulative proportions of virological failure, non-resistance and second-line treatment at the end of the five-year modelling period for each scenario. In the routine monitoring (RM) scenario, 19.8% of patients on first-line therapy fail virologically during their first five years and are switched to second-line therapy. In the resistance testing (RT) and limited testing (LT) scenarios, slightly more (20.2%) patients fail virologically, with the extra 0.4% failures reflecting that fewer patients are switched to second-line therapy each cycle. These patients are still on first-line therapy at the end of the cycle and thus remain in the exposure pool for first-line failure in the following cycle. The cumulative number failing by the end of five years is thus slightly higher.

**Table 3:** Proportions with virological failure, non-resistance, and second line switch, by strategy (1000-patient Markov cohort analysis)

Strategy	Proportion of cohort with virological failure	Proportion of virological failures identified as non-resistant	Proportion of cohort switched to second-line therapy
Routine monitoring (RM)	19.8%	0.0%	19.8%
Resistance testing (RT)	20.2%	16.8%	16.7%
Limited testing (LT)	20.2%	11.4%	17.8%

In the RT scenario, 16.8% of the failures do not have any mutations and are kept on first-line treatment, resulting in a somewhat smaller proportion (16.7%) of the original cohort ending up on second-line therapy by the end of five years. In the LT strategy, in which resistance testing is done only if the patient with virological failure started ART three years or less before, 17.8% of patients are switched to second line.

**Figure 2:**
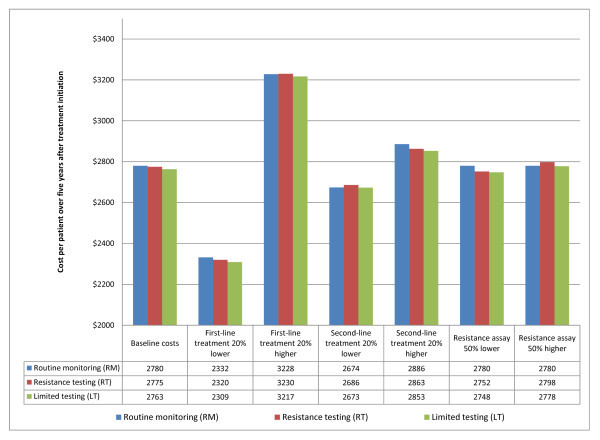
**Results of baseline and sensitivity cost analyses**.

Cost estimates from the baseline analysis and sensitivity analyses are shown in Figure 2. In the RM scenario, the total average cost per patient over the five-year period is $2780 ($556/year) (95% SI $2761-2800). The total average cost per patient for the RT scenario is $2775 ($555/year) (95% SI $2755-2795), almost identical to that of the RM scenario. In the LT scenario, the total average cost per patient over five years is $2763 ($553/year) (95% SI $2743-2783), slightly less than the cost of the other scenarios.

Using the baseline cost values, the total costs of all three scenarios are almost the same. The sensitivity analysis illustrated in Figure 2 shows that the baseline findings change little in response to the modelled changes in unit costs. The LT strategy remains very slightly less expensive than the other strategies across all changes in cost parameters. The cost of the LT strategy is nearly identical to that of the RM strategy if the cost of second-line therapy falls or the cost of a resistance test increases, however. The ranking of the full RT strategy and the RM strategy is sensitive to the cost parameter values, but the differences between them remain modest in magnitude.

## Discussion

Using recent parameter estimates from a large South African public sector treatment site, a strategy that incorporates resistance testing into decisions on whether to switch patients with first-line virologic failure to second-line therapy is potentially cost neutral. It helps identify patients failing for reasons other than viral resistance; it conserves future treatment options for these patients and spares them the additional toxicity burden of second-line drugs; and it generates better information about emerging drug-resistance patterns.

The analysis presented here has a number of limitations. It is based on patient-level data from a single treatment site, which may not be representative of all sites in South Africa, and estimates parameters under treatment guidelines that have since been revised. Rates of non-resistance among virological failures by duration on ART are based on very small patient numbers and drawn from a single-site sample that may or may not be representative of all patients failing virologically. Due to data limitations, we were not able to take into account variation in the timing of viral load tests relative to the emergence of resistance or the possibility that the percentage of virologic failures that occur without resistance may vary by sub-population.

Mortality and loss to follow up are also not taken into account. Treatment cost estimates reflect drug choices and prices in effect at the time of the analysis; drug prices vary widely and change frequently; and a substantial alteration in the relative costs of first- and second-line therapy could have a large impact on model results. The cost of performing a resistance test may also change with technological advances and laboratory scale.

## Conclusions

Despite its limitations, this analysis suggests that the net cost of incorporating resistance testing into treatment guidelines would not be prohibitive, and it provides a model for examining this cost under different input assumptions and treatment strategies. In countries that use viral loads to guide treatment decisions and have the capacity to conduct resistance testing at a cost similar to that cited here, incorporating it into treatment guidelines should be considered, and a follow-on analysis assessing the net benefits of this strategy, as well as its net costs, should be undertaken.

## Competing interests

The authors declare that they have no competing interests.

## Authors' contributions

SR, MF, IS and WS conceived of the analysis. SR designed the model, performed the analysis, and drafted the manuscript. LL and MF estimated the parameters. IS provided the data. All authors read, commented on and approved the final manuscript.
